# An isolated cellulolytic *Escherichia coli* from bovine rumen produces ethanol and hydrogen from corn straw

**DOI:** 10.1186/s13068-017-0852-7

**Published:** 2017-06-24

**Authors:** Jian Pang, Zhan-Ying Liu, Min Hao, Yong-Feng Zhang, Qing-Sheng Qi

**Affiliations:** 1 School of Chemical Engineering, Inner Mongolia University of Technology, Hohhot, 010051 Inner Mongolia China; 2 Institute of Coal Conversion & Cyclic Economy, Inner Mongolia University of Technology, Hohhot, 010051 Inner Mongolia China; 3 State Key Laboratory of Microbial Technology, Shandong University, Jinan, 250100 China

**Keywords:** Rumen, Isolation, Cellulolytic *Escherichia coli*, Cellulase, Ethanol

## Abstract

**Background:**

Lignocellulosic biomass is the most abundant resource on earth. Lignocellulose is mainly composed of cellulose, hemicelluloses, and lignin. The special construction of three kinds of constituents led to the prevention of effective degradation. The goal of this work was to investigate the great potentials of bovine rumen for novel cellulolytic bacterial isolation, which may be used for chemicals and biofuel production from lignocellulose.

**Results:**

A cellulolytic strain, ZH-4, was isolated from Inner Mongolia bovine rumen. This strain was identified as *Escherichia coli* by morphological, physiological, and biochemical characteristics and 16S rDNA gene sequencing. The extracellular enzyme activity analysis showed that this strain produces extracellular cellulases with an exoglucanase activity of 9.13 IU, an endoglucanase activity of 5.31 IU, and a β-glucosidase activity of 7.27 IU at the pH 6.8. This strain was found to produce 0.36 g/L ethanol and 4.71 mL/g hydrogen from corn straw with cellulose degradation ratio of 14.30% and hemicellulose degradation ratio of 11.39%.

**Conclusions:**

It is the first time that a cellulolytic *E. coli* was isolated and characterized form the bovine rumen. This provided a great opportunity for researchers to investigate the evolution mechanisms of the microorganisms in the rumen and provided great chance to produce biofuels and chemicals directly from engineered *E. coli* using consolidated bioprocess.

## Background

Lignocellulosic biomass is the most abundant carbohydrate in nature, and also one of the most important renewable resources [1]. Lignocellulose consists mainly of cellulose (35–50%), hemicellulose (25–30%), and lignin (25–30%) [2]. As the main component of lignocellulose, cellulose is made up of linear chains of 1,4-β-linked glucosyl residues and enwrapped by hemicellulose and lignin, which prevents the effective degradation [3, 4].

Among cellulose degradation technology, enzymatic hydrolysis is an environmentally friendly and effective technique. However, the lack of enzymes or microbes which can efficiently deconstruct plant polysaccharides represents a major bottleneck. The cellulolytic microorganisms inhabit in a wide range of niche, such as soil, compost piles, decaying plant materials, rumens, sewage sludge, forest waste piles, and wood processing plants [5]. Therefore, a lot of researchers have tried to isolate high efficient cellulase-producing microorganisms worldwide in different niches [6, 7]. Although fungi and actinomyces played a major role in the degradation of plant materials in natural niche, cellulolytic bacteria have their unique properties compared to fungi and actinomyces, which provides us a variety of cellulase selections for the biofuel and biorefining industry [8, 9]. So far, different kinds of cellulolytic bacteria were isolated from different environments, such as anaerobic bacteria *Acetivibrio*, *Bacteroides*, *Clostridium*, *Ruminococcus* and aerobic bacterium *Bacillus* sp., *Cellulomonas*, *Cellvibrio*, *Microbispora*, *Thermomonospora* sp., *Cellulomonas* sp., *Pseudomonas*, *Nocardiopsis* sp., *Cellulomonas* sp. [9]. Aerobic bacteria secrete individual, extracellular enzymes with binding modules for various cellulose conformations and the enzymes act in synergy to degrade cellulose. However, anaerobic bacteria produce a unique extracellular multi-enzyme complex, cellulosome, to hydrolyze cellulose effectively [10]. It was supposed that the latter showed a higher degradation efficiency and conversion rate for lignocellulose [11]. The microorganisms in rumen anaerobic ecosystem are more diverse and many enzymatic activities are involved, such as cellulase, xylanase, β-glucanase, pectinase, amylase, protease, and so on [12–14]. However, few members of this complex community have been isolated and cultivated in vitro because of the sensitivity to oxygen and special nutritional requirements [15]. To avoid the difficulties in isolation and cultivation, some uncultured methods based on molecular biology have been carried out, such as metagenomics, genomic library, full-cycle rRNA analysis, and fingerprinting [16]. Metagenomics is applied as a high-throughput next-generation sequencing technologies to characterize microorganism communities in the absence of culture [17]. Biomass-degrading genes and genomes have been focused to investigate the function and degradation mechanisms of these ruminal cellulolytic microorganisms by metagenomics [18]. Analyses of metagenomics and the 16S pyrotag library of the rice straw-adapted microbial consortia showed that the phylum *Actinobacteria* was the main group, and approximately 46.1% of CAZyme genes were from actinomycetal communities [19]. A novel encoded bifunctional xylanase/endoglucanase gene, *RuCelA*, was cloned from the metagenomics library of yak rumen microorganisms [20]. However, pure microorganism can’t be obtained by metagenomics, and thus the intensive studies on evolutionary process and characteristics of microorganisms were hindered. As a result, researchers have tried to directly isolate cellulolytic microorganisms from rumen. For example, *Enterobacter cloacae* WPL 214 was isolated from bovine rumen fluid waste of Surabaya, Indonesia and *Sphingobacterium alimentarium* was isolated from buffalo rumen fluid [21, 22]. A novel *Clostridiaceae* (AN-C16-KBRB) with bifunctional endo-/exo-type cellulase was isolated from the bovine rumen at Jeongeup in Korea, while *Treponema* JC4 was isolated from bovine rumen in the semiarid tropics of Australian [23, 24].

In this study, an *Escherichia coli* strain with cellulose-degrading and hemicellulose-degrading activity was isolated from Inner Mongolia bovine rumen for the first time. Three kinds of cellulase activities, exoglucanase, endoglucanase, and β-glucosidase activities were detected in the fermentation supernatant under the condition of neutral buffered solution, which indicated that this strain has special properties with regards to cellulose degradation in comparison to normal *E. coli*. The production of ethanol and hydrogen from corn straw was also investigated.

## Results

### Screening of cellulolytic bacteria from bovine rumen

**Fig. 1:**
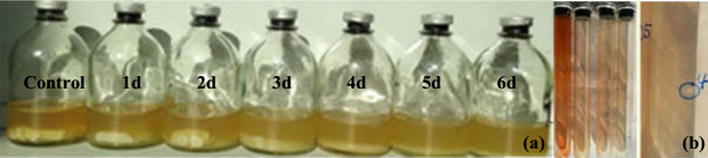
The result of screening (**a**) and verification (**b**)

The sample was taken from bovine rumen and was enriched in a medium with Whatman filter paper as sole carbon source. After 6 days, the filter paper was found to be degraded efficiently, indicating the presence and enrichment of cellulolytic microorganisms in the sample (Fig. 1a). Then the samples were transferred 3 times to remove the non-cellulose-degrading microorganisms. The enriched samples were diluted and then spread in strict anaerobic Hungate roll tubes that contain the cellulose and Congo red. The anaerobic cellulolytic microorganisms should grow under strict anaerobic condition and degrade cellulose to form a transparent zone. The cellulose-degrading strains MA-1, WH-2, PJ-3, ZH-4, HM-5, which formed a clear zone in the tube, were picked, respectively (Fig. 1b). Strain ZH-4, which has the biggest ratio of transparent circle and a colony diameter of 6.8 mm/mm, was further purified with Hungate roll tubes and was used for detailed characterization and study.

### Identification and characterization of isolated cellulose-degrading strain

**Fig. 2:**
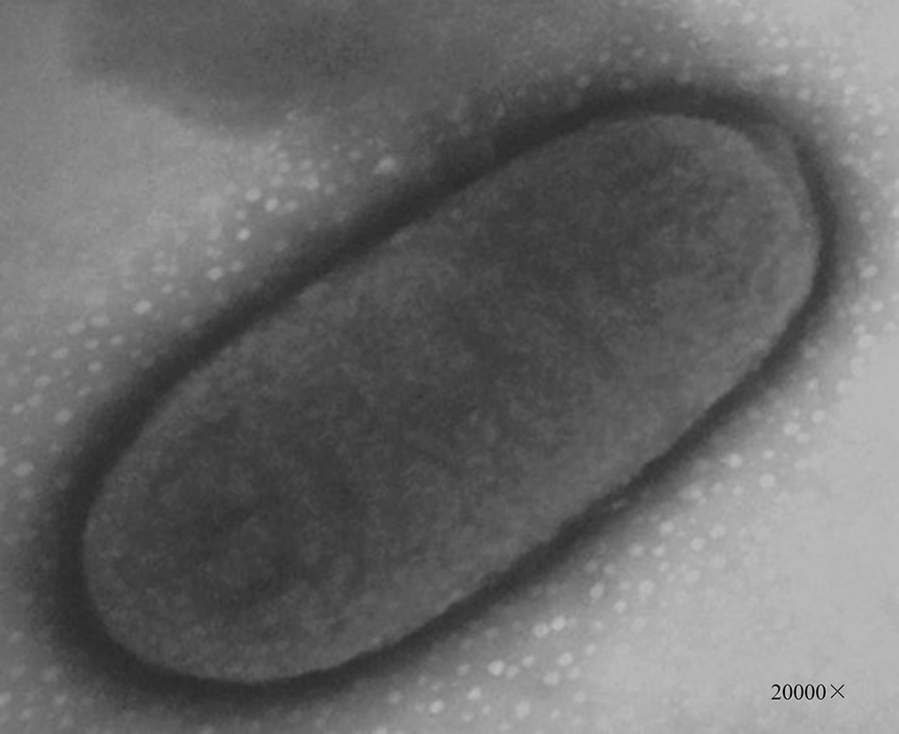
Transmission electron micrograph of ZH-4

The colony characteristics of ZH-4 were round, smooth, wet, milkness. The edge of the colony is regular. Gram staining showed that this strain belonged to Gram-negative bacterium with a size of 0.87 × 1.63 μm. The transmission microscope is shown in Fig. 2.

**Fig. 3:**
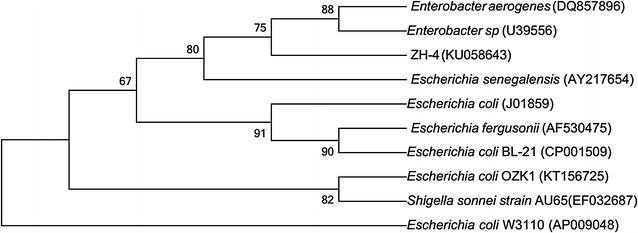
Phylogenetic tree of 16S rDNA gene sequence. The evolutionary history was inferred using the neighbor-joining method. The bootstrap consensus tree inferred from 1000 replicates was taken to represent the evolutionary history of the taxa analyzed. Branches corresponding to partitions reproduced in less than 50% bootstrap replicates were collapsed. The percentage of replicate trees in which the associated taxa clustered together in the bootstrap test (1000 replicates) was shown next to the branches. The evolutionary distances were computed using the Maximum Composite Likelihood method and were in the units of the number of base substitutions per site. Evolutionary analyses were conducted using MEGA 7 software

ZH-4 presents an optimum growth temperature of 37 °C and optimum pH of 7.0 (data not shown). The physiological and biochemical characteristics of ZH-4 were analyzed (Table 1), which indicated that this strain may belong to enteric bacteria, *Escherichia* *coli*. Since common *E. coli* did not degrade cellulose, to further confirm our result, the strain ZH-4 was identified again with Biolog MicroPlate reader (ICN Flow Titertek^®^ Multiskan Plus, Version 2.03, LabSystems, Finland), which also showed the highest similarity to *E.* *coli*. Then we extract and sequenced the 16S rDNA (1444 bp, Genbank accession number KU058643) of strain ZH-4 and blasted using BLAST search program in the GenBank of NCBI, which revealed 99% sequence identity with *E. coli*. The relationship between the strain ZH-4 and the most close taxonomic species based on 16S rDNA sequences was described in the phylogenetic tree (Fig. 3). The genome sequencing results showed that ZH-4 belonged to *E. coli*. The genome size was about 5.28 Mb with GC% 50.69. NR annotation also matched with *E. coli.* Strain ZH-4 had been preserved in China General Microbiological Culture Collection Center (CGMCC). The preservation number of CGMCC was No. 12427.

**Table 1:** Physiological and biochemical characteristics of ZH-4

Characteristics	Reaction		Characteristics	Reaction	
	ZH-4	Control group		ZH-4	Control group
Glucose	+	+	Mannitol	+	+
Fructose	+	+	Dulcite	+	‒
Arabinose	+	+	Glycerol	+	+
Raffinose	‒	+	Gelatin	‒	‒
Lactose	+	+	Salicylic acid	+	+
Melibiose	+	+	Esculin	+	+
Xylose	+	+	Urea	‒	‒
cellobiose	‒	‒	hydrogen sulfide	‒	‒
Rhamnose	+	+	Nitrate	+	+
Maltose	+	+	Citrate	‒	‒
Sucrose	‒	‒	Phenylalanine deaminase	‒	‒
Galactose	+	+	Ornithine decarboxylase	+	+
Starch	‒	‒	Lysine decarboxylase	+	+
Peptone water	+	+	Motility	+	+
Sorbitol	+	‒	Optimal pH for growth	7.0	
Optimal temperature for growth	37 °C				

+ positive reaction; − negative reaction

All these experiments confirmed that the strain ZH-4 is enteric bacteria, *E. coli*. On the other hand, our experiment results also clearly indicated that ZH-4 can degrade cellulose, while the typical *E. coli* strain MG1655 and W3110 cannot at the same condition (data not shown). To further confirm this phenomenon, the enzymatic activities of the fermentation fluid with regard to the cellulose degradation were analyzed.

### Confirmation of the cellulolytic property by enzymatic assay

**Fig. 4:**
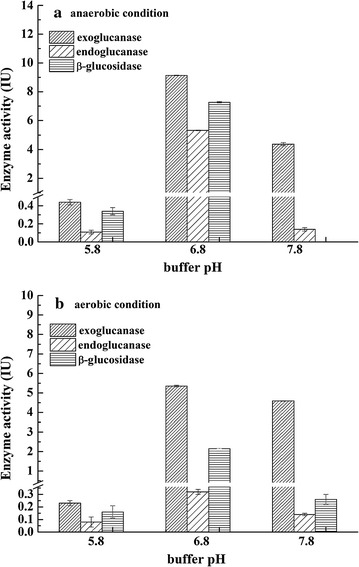
Cellulase activity under anaerobic condition with different pH (**a**); cellulase activity under aerobic condition with different pH (**b**)

**Fig. 5:**
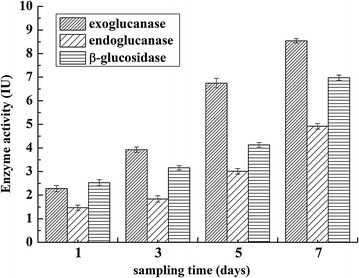
Cellulase activity with cultivation time

Since no cellulosome structure was found on the surface of bacteria via TEM, the endoglucanase, exoglucanase, and β-glucosidase activities in fermentation supernatant were analyzed both under aerobic and anaerobic conditions (Fig. 4). From results, we found that strain ZH-4 possesses all endoglucanase, exoglucanase, and β-glucosidase activities, which explained why this strain can degrade cellulose efficiently. The extracellular enzyme activities increased along with the cultivation time (Fig. 5). The highest enzyme activities, an exoglucanase activity of 8.54 IU, an endoglucanase activity of 4.92 IU, and a β-glucosidase activity of 6.98 IU were found on the 7th day. Meanwhile, we found that the enzyme activities are higher under anaerobic condition than that under aerobic condition that also fits to the rumen environments. The highest enzyme activities appeared at neutral conditions of pH 6.8. At this condition, an exoglucanase activity of 9.13 IU, an endoglucanase activity of 5.31 IU, an β-glucosidase activity of 7.27 IU were found under anaerobic condition at pH 6.8, while an exoglucanase activity of 5.35 IU, an endoglucanase activity of 0.32 IU, and a β-glucosidase activity of 2.15 IU were found under aerobic condition. At pH 5.8, all enzyme activities were low, while at pH 7.8 the exoglucanase was still quite high. This indicated that the exoglucanase is alkali-resistant. The enzyme activity assay demonstrated that ZH-4 do generate soluble cellulose-degrading enzymes. However, the enzyme generation and secretion mechanism shall be investigated further.

### Production of ethanol and hydrogen from corn straw

**Fig. 6:**
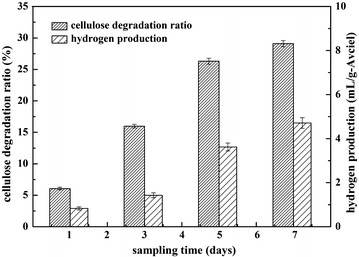
Fermentation of ZH-4 with Avicel as substrate

To evaluate the fermentation performance and biofuels and chemicals production potential from biomass of ZH-4, crystallized cellulose Avicel was initially tried. After cultivation optimization, the fermentation condition was determined to be 37 °C and pH 6.8 under anaerobic conditions. At this condition, the fermentation broth became muddy, the color changed dark, and the amount of crystallized cellulose Avicel was reduced gradually; a possible explanation of this phenomenon was that the crystallized cellulose Avicel had been solubilized by the strain ZH-4. Cellulose degradation ratio, ethanol titer, and hydrogen accumulation were analyzed (Fig. 6). The cellulose degradation ratio (utilization in absolute concentration) was 6.06% (0.30 g/L), 15.99% (0.79 g/L), 26.31% (1.30 g/L), 29.10% (1.44 g/L), and the hydrogen production was 0.83, 1.43, 3.62, 4.71 mL/g-Avciel, respectively, after 1 to 7 days of cultivation. The highest hydrogen accumulation was observed on 7th day. However, ethanol was not detected. This result proved that strain ZH-4 can produce biofuels directly from cellulose.

**Fig. 7:**
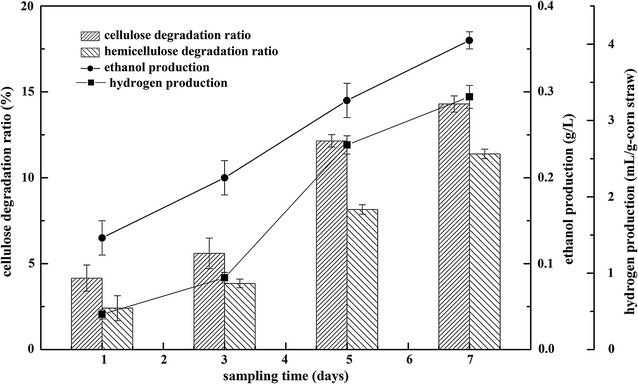
Fermentation of ZH-4 with Corn straw as substrate

To further evaluate the actual performance of the strain from biomass, the corn straw, which consists of 36.3% glucan and 14.9% xylan and was used as substrate to investigate the ethanol and hydrogen production potential (Fig. 7). The corn straw was found to be efficiently degraded in the presence of only strain ZH-4, although hemicellulose enzyme activity was not detected in the pre-experiments (data not shown). The cellulose degradation ratio (utilization in absolute concentration) was 4.16% (0.23 g/L), 5.60% (0.31 g/L), 12.15% (0.66 g/L), 14.30% (0.78 g/L) and the hemicellulose degradation ratio was 2.41% (0.05 g/L), 3.85% (0.09 g/L), 8.16% (0.18 g/L), 11.39% (0.26 g/L) after 1, 3, 5, and 7 days of fermentation. The color of fermentation broth showed to be more yellow after 5 days of cultivation, which was probably related to the biological decomposition of corn straw. According to the analysis of reducing sugars, the corn straw was continuously degraded throughout the 7-day period. The final ethanol titer was 0.36 g/L and the final hydrogen production was 3.31 mL/g-corn straw with 15.32% cellulose and 11.39% hemicellulose were degraded after 7 days of cultivation. As a new microbial resource, this strain can be optimized to produce more ethanol and H_2_ through metabolic engineering and process optimization.

## Discussion

Many previous isolated cellulolytic bacteria from different habitats are strictly anaerobic, for example, *Bacteroides*, *Clostridium*, *Ruminococcus* [9]. It was the first time that a cellulolytic *E. coli* was found in the bovine rumen. Since *E. coli* was previously not found with regards to its cellulose degradation capability [25], several purification steps were performed to ensure the purity of the isolates. The anaerobic cellulose-based screening also removed the non-cellulolytic bacteria and many possible contaminations. Meanwhile, the isolated ZH-4 was again detected with respect to their enzyme activity for confirmation of our result. 16S rDNA and genome sequencing analysis not only confirmed the purity of the isolate but also revealed 99% sequence identity with *E. coli*.

In previous studies, a cellulase-producing *Enterobacter cloacae* WPL 214 was isolated from bovine rumen fluid waste. The endoglucanase activity of 0.009 U/mL, exoglucanase of 0.13 U/mL, and cellobiase of 0.10 U/mL were obtained at optimum temperature of 35 °C and optimum pH of 5 [21]. Cellulase-producing *Sphingobacterium alimentarium* isolated from buffalo rumen fluid had an extracellular cellulase activity of 1.363 IU/mL [22]. Besides, a microaerophilic *bacillus licheniformis* with a high cellulose-degrading activity was also isolated from bovine rumen. This indicated that even in bovine rumen, microaerobic or facultative bacteria also exist. It had been reported that rumen bacteria degraded hemicellulose from bromegrass by pure cultures of *Bacteroides succinogenes* A3c, S-85, and *Ruminococcus albus* 7; the hemicellulose degradation ratio were 54.0, 77.3, and 60.9%, respectively [26]. The rumen bacteria were known to produce ethanol and hydrogen via fermenting lignocellulosic materials under anaerobic condition [27]. For *Ruminobacter albus* and *Ruminococcus flavefaciens*, hydrogen and ethanol are major end metabolites under anaerobic conditions [7]. The rumen anaerobic fungus *Neocallimastix frontalis* also produces ethanol and hydrogen as end products [28]. It is well known that wild-type *Escherichia coli* neither produces endogenous cellulose degradation enzymes nor secretes heterologous cellulases due to its poor secretory capacity, and there is no report that wild-type *E. coli* expresses cellulase and possesses cellulose-degrading and hemicellulose-degrading ability [29].

*Escherichia coli* often was used as a cell factory for the production of biofuels and chemicals on account of its many available expression and regulation tools [30]. Native *E. coli* was to produce biochemicals by expressing heterologous cellulases [29, 31]. However, the novel *E. coli*, ZH-4, not only had a self-contained complete cellulase system but also secreted these enzymes outside the cells. And also, the endoglucanase genes (Genbank accession number KY965823), β-glucosidase genes (Genbank accession number KY965824), and hydrogenase genes (Genbank accession number KY965825) were identified in the draft genome. The genes that are responsible for the production of cellulases and hydrogen had been found. This new characteristics are supposed to be evolved from its special niche in the rumen. Hence, we speculated that the cellulase genes may come from horizontal gene transfer in the rumen ecology system. The cellulase genes in *Enterococcus faecalis* were thought to be from horizontal gene transfer [32]. The gene transfer of an endoglucanase (celA) from *Fibrobacter succinogenes* of rumen bacteria to the rumen fungi *Orpinomyces joymii* was also reported [33]. These examples indicated that the rumen, as a special environment, has the special condition to obtain the unique genes from other bacteria or have special mechanisms to regulate gene expression system. However, we also cannot exclude the possibility that the potential cellulase gene in wild-type *E. coli* was activated due to some reasons. To demonstrate that, the complete sequencing is to be done, and regulation analysis should be performed to strain ZH-4. Since *E. coli* was normally used as cell factory, it may be of great interest for the production of biofuels and chemicals using the consolidated process or single cell refinery from biomass [34].

## Conclusions

In this study, a cellulose-degrading *E. coli* strain ZH-4 was isolated from the rumen of Inner Mongolia bovine, and was identified as a new isolate of *Escherichia* *coli*. The strain showed extracellular cellulase activity and can produce ethanol and hydrogen from cellulose and corn straw, indicating the great potential application of the strain. Further metabolic and regulating investigation of the strain may reveal the secretion mechanisms of the enzymes in ZH-4.

## Methods

### Materials and Sample collection

All the chemicals used were of molecular biology or analytical grade. Corn straw (corn type: pioneer) was collected in mature period from the suburbs of Hohhot City, China. The whole corn straw was collected, cut into 2–3 cm in length, and then crushed by crusher and sieved by a 40-mesh sieve for use.

Rumen contents were collected from three Inner Mongolia bovine. Samples were collected and filtered by four layers of gauze into 150 mL sterile and N_2_-gassed glass containers with screw-top sealable metal lids and kept in 37 °C, and then brought to the laboratory for use.

### Enrichment of cellulolytic bacteria

A 15% (v/v) inoculum of rumen samples were inoculated in enrichment medium A with microcrystalline cellulose as carbon source. Then, the serum bottles were incubated at 37 °C and 180 rpm for 6 days. After the first enrichment, 15% (v/v) inoculum of culture was inoculated in enrichment medium A with Whatman filter paper as carbon source replacing microcrystalline cellulose. The cellulose-degrading strains were enriched by three successive 6-day subcultures. The enrichment medium A was as follows: basic medium (500 mL): NaHCO_3_ 5.0 g, peptone 1.0 g, yeast extract 1.0 g; Mineral A (165 mL): KH_2_PO_4_ 0.3 g, (NH_4_)_2_SO_4_ 0.3 g, NaCl 0.6 g, CaCl_2_·2H_2_O 0.04 g, MgSO_4_·7H_2_O 0.058 g; Mineral B (165 mL): K_2_HPO_4_·3H_2_O 0.396 g, cell-free rumen fluid 170 mL, l-cysteine hydrochloride 1.5 g, microcrystalline cellulose 5.0 g. The cell-free rumen fluid was obtained by the following procedure: rumen fluid was taken from the rumen of the Inner Mongolia bovine, and then filtered by 4 layers of gauze. The filtrate was centrifuged by 5000 rpm for 15 min at the first time and 15,000 rpm for 30 min for the second time.

### Screening of cellulolytic bacteria

Congo red staining method was used for strain screening. 10-fold dilution of log-phase cells after three successive enrichment cultures were inoculated into screening Hungate roll tubes (Medium B), and subsequently prepared by rapidly rotating inoculated tubes under an ice bath. Then, Hungate roll tubes were incubated at 37 °C incubator and observed for the characteristic of colonies and transparent zones regularly. The colonies that formed transparent zones in the Carboxymethylcellulose (CMC) agar were picked out. The pure strain was confirmed through observing the colony and cell morphology. The single colonies were preserved. The impurity colonies were purified by roll tube isolation. Some representative cellulose-degrading bacteria were successfully isolated by this isolation procedure. The pure isolate with the biggest ratio of transparent circle and colony diameter among others was picked for further verification of cellulose degradation ability. Medium B was composed of of basic medium, Mineral A, Mineral B, cell-free rumen fluid 170 mL, l-cysteine hydrochloride 1.5 g, CMC 5.0 g, agar 20.0 g, Congo red dye 0.4 g. All of the medium were dispensed under N_2_ gassing into serum bottle or Hungate roll tubes with aluminum crimp and flanged butyl stoppers and then were autoclaved [35]. All operations must be under anaerobic conditions. The isolated cellulolytic strains were stored at −70 °C.

### Identification of strains

The characteristic of colony which grow on the solid medium was recorded and cell morphology was observed through a microscope (biological microscope XS-212, China) and a transmission electron microscope (TEM H-700, Hitachi, Japan) with accelerating voltage 75 kV and magnification of 20,000×. Physical and biochemical parameters were identified with the kit of microbial biochemical identification and compared according to Bergey’s Manual of Systematic Bacteriology [36]. Gram staining was carried out according to the Manual of Methods for General Bacteriology [37].

The purified bacterium was identified using the Biolog GEN III MicroPlate™, which contains 71 kinds of carbon source, 23 types of chemical sensitivity assays and positive and negative controls. Single colony from AN plates were transferred onto Biolog universal growth agar and incubated at 37 °C for 24 h. Colonies were picked by a sterile moistened Biolog cotton swab, and then were suspended in sterile inoculating fluid. The concentration was adjusted to match Biolog GEN III turbidity standards. The isolate was inoculated on a separate Biolog GEN III MicroPlate (Biolog Inc., Hayward, CA). After incubating at 37 °C for 24 h, the isolate was determined by MicroStation 2 Reader. The isolate will be identified if the Similarity (SIM) >0.5 after 24 h. SIM value referred to the similarity of the experimental result with corresponding data in the Biolog database.

## 16S rDNA sequencing, phylogenetic analysis, and genome sequencing

Molecular biology method is a more accurate method for strain identification. 16S rDNA gene sequencing is a widely used, effective, and simple technique. The genomic DNA was extracted from the cultures in exponential growth phase. 16S rDNA sequencing was carried out through the procedures of DNA extraction, PCR amplification, cloning, and sequencing in Sangon Biotech (Shanghai, China) [38]. The 16S rDNA gene sequences were compared with others using BLAST program. The phylogenetic tree was constructed based on a neighbor-joining algorithm with the MEGA version 7.0 software and the bootstrap consensus tree inferred from 1000 replicates was taken to represent the evolutionary history of the taxa analyzed [39]. The draft genome was completed by Novogene company (China).

### Enzymatic assay

Two percentage (v/v) seed was inoculated in medium D (peptone 10 g/L, yeast extract 10 g/L, NaCl 10 g/L, microcrystalline cellulose 5 g/L), with microcrystalline cellulose as carbon source, and then incubated at 37 °C for 7 days under anaerobic and aerobic conditions, respectively. The fermentation broth was centrifuged with 8000 rpm for 5 min at 4 °C and the supernatant was used as a source of crude enzyme.

Three kinds of extracellular cellulase (exoglucanase, endoglucanase, and β-glucosidase) activities were measured by determining the amount of reducing sugar released from microcrystalline cellulose, CMC, and salicin according to that described by Wood TM [40]. The cellulolytic enzyme activities were determined in different pH value buffer solutions (5.8, 6.8, 7.8) under anaerobic and aerobic conditions, respectively. All spectrophotometric data were measured using a UV–Vis spectrophotometer (UV759; China). One unit (IU) of enzymatic activity is defined as the amount of enzyme that releases 1 μmol reducing sugars (measured as glucose) per mL fermentation supernatant per minute.

### Fermentation of ZH-4 using cellulose and hemicellulose

Thirty milliliter medium A containing corn straw (15 g/L) or microcrystalline cellulose (5 g/L) as the carbon source was prepared, respectively, in 100 mL serum bottle. A 5% (v/v) inoculum isolates were inoculated in these medium under anaerobic conditions, respectively. Then, the serum bottles were incubated at 37 °C and 180 rpm for 1, 3, 5, and 7 days. The gas was collected using an airtight bag for GC–MS analysis that produced in the anaerobic serum bottle. The degradation ratio of cellulose and hemicellulose was measured by quantitative saccharification as described by Shao et al. [41]. The fermentation broth was centrifuged by 8000 rpm for 10 min. The supernatant was acidized by 10% sulfuric acid solution and then filtered by 0.22 μm filter membrane. 1 mL 72% H_2_SO_4_ was added to 28 mL supernatant and autoclaving at 121 °C for 60 min. After spinning filter, concentrations of ethanol and sugars were obtained using a Waters HPLC system. Conversions were calculated as a percentage of originally present glucan and xylan solubilized, based on the analysis of residual solids. Glucan solubilization and xylan solubilization were calculated according to Eq. 1 and Eq. 2. Avicel was composed of 99% glucan. Avicel degradation ratio was calculated by glucan solubilization according to Eq. 1.

1 $${\text{glucan solubilization }} = \left( {1 - \frac{{\frac{{\mathop C\nolimits_{{\text{glucose}}} \times 162}}{180} \times \mathop V\nolimits_{2} }}{{\mathop C\nolimits_{i} \times \mathop V\nolimits_{1} \times a}}} \right) \times 100\%$$

*C*_glucose_: the concentration of glucose in the hydrolysate of residual Avicel, g/L. *C*_*i*_: the initial concentration of Avicel or Corn straw, 5 g/L for Avicel, or 15 g/L for corn straw. *V*_*1*_: the broth volume after inoculation, L. *V*_*2*_: the initial culture medium, L. *a*: the percentage of glucan in Avicel or Corn straw. 99% in Avicel and 36.3% in Corn straw.

The 36.3% glucan and 14.9% xylan in the corn straw were determined according to the methods as reported by the National Renewable Energy Laboratory (NREL) [42].

Corn straw degradation ratio was calculated by glucan solubilization (Eq. 1) and xylan solubilization (Eq. 2).

2 $${\text{xylan solubilization }} = \left( {1 - \frac{{\frac{{\mathop C\nolimits_{{\text{xylan}}} \times 132}}{150} \times \mathop V\nolimits_{2} }}{{\mathop C\nolimits_{i} \times \mathop V\nolimits_{1} \times b}}} \right) \times 100\%$$

*C*_xylan_: the concentration of xylan in the hydrolysate of residual corn straw. *C*_*i*_: the initial concentration of Corn straw, 15 g/L. *V*_*1*_: the broth volume after inoculation, L. *V*_*2*_: the initial culture medium, L. *b*: the percentage of xylan in corn straw, 14.9%.

### Analytical methods

The concentrations of ethanol and sugars in supernatant were analyzed by HPLC Aminex HPX-87H column (300 mm × 7.8 mm id, 9 μm). Aqueous sulfuric acid solution (8 mM) as a mobile phase was pumped at the rate of 0.5 mL/min and the column and refractive index detector temperatures are both 40 °C. The hydrogen accumulation was measured by a GC–MS (model SP-6800A, column model TDX-1) using the method of Li et al. [43]. The temperatures of column, heater, and detector temperatures were 160, 180, and 180 °C, respectively.

### Statistical analysis

All experiments were conducted in triplicate and the data were presented as mean values ± standard deviation. An analysis of variance (ANOVA) for the obtained results was carried out using SAS 9.2 software (SAS INSTITUTE INC, North Carolina, USA).

## Abbreviations

CMC: carboxymethylcellulose
CGMCC: China General Microbiological Culture Collection Center
TEM: transmission electron microscope
SIM: Similarity
HPLC: high performance liquid chromatography
GC-MS: gas chromatography–mass spectrometer
ANOVA: analysis of variance
